# *Mycoplasma genitalium* Infection and Chronic Inflammation in Human Prostate Cancer: Detection Using Prostatectomy and Needle Biopsy Specimens

**DOI:** 10.3390/cells8030212

**Published:** 2019-03-02

**Authors:** Makito Miyake, Kenta Ohnishi, Shunta Hori, Akiyo Nakano, Ryuichi Nakano, Hisakazu Yano, Sayuri Ohnishi, Takuya Owari, Yosuke Morizawa, Yoshitaka Itami, Yasushi Nakai, Takeshi Inoue, Satoshi Anai, Kazumasa Torimoto, Nobumichi Tanaka, Tomomi Fujii, Hideki Furuya, Charles J. Rosser, Kiyohide Fujimoto

**Affiliations:** 1 Department of Urology, Nara Medical University, 840 Shijo-cho, Kashihara, Nara 634-8522, Japan; kenzmedico0912@yahoo.co.jp (K.O.); horimaus@gmail.com (S.H.); sayuri3@naramed-u.ac.jp (S.O.); tintherye@gmail.com (T.O.); tigers.yosuke@gmail.com (Y.M.); y.itami.324@gmail.com (Y.I.); nakaiyasusiuro@live.jp (Y.N.); you1513tt@yahoo.co.jp (T.I.); sanai@naramed-u.ac.jp (S.A.); torimoto@nmu-gw.naramed-u.ac.jp (K.T.); sendo@naramed-u.ac.jp (N.T.); kiyokun@naramed-u.ac.jp (K.F.); 2 Department of Microbiology and Infectious Diseases, Nara Medical University, 840 Shijo-cho, Kashihara, Nara 634-8522, Japan; akiyo@naramed-u.ac.jp (A.N.); rnakano@naramed-u.ac.jp (R.N.); yanohisa@naramed-u.ac.jp (H.Y.); 3 Department of Diagnostic Pathology, Nara Medical University, 840 Shijo-cho, Kashihara, Nara 634-8522, Japan; fujiit@naramed-u.ac.jp; 4 University of Hawaii Cancer Center, Clinical and Translational Research, Honolulu, HI 96813, USA; hfuruya@hawaii.edu (H.F.); crosser@cc.hawaii.edu (C.J.R.)

**Keywords:** *Mycoplasma genitalium*, human papillomavirus, prostate cancer, inflammation, polymerase chain reaction

## Abstract

The evidence of association between sexually transmitted infection and prostatic inflammation in human prostate cancer (PCa) is limited. Here, we sought to examine the potential association of prostatic infection with the inflammatory environment and prostate carcinogenesis. We screened surgical and biopsy specimens from 45 patients with PCa against a panel of sexually transmitted infection-related organisms using polymerase chain reaction and examined the severity of intraprostatic inflammation by pathologic examination. Among tested organisms, the rate of *Mycoplasma genitalium* (Mg) infection was significantly different between the prostate cancer cohort and benign prostate hyperplasia (BPH) cohort (*P* = 0.03). Mg infection in the surgical specimens was associated with younger patients. The rate of extensive disease (pT2c–3b) was higher in Mg-positive patients than in Mg-negative patients (*P* = 0.027). No significant correlation was observed between Mg infection status and the grade of intraprostatic inflammation. The detection sensitivity of biopsy specimens was 61% for Mg and 60% for human papillomavirus (HPV)18, indicating possible clinical application of this material. A comprehensive understanding of the correlation between the urogenital microbiome and inflammation would facilitate the development of strategies for PCa prevention. Further studies are required to explore its clinical utility in recommendations of early re-biopsy, close follow-up, and treatment by antibiotics.

## 1. Introduction

Approximately one in every five malignancies can be attributed to infectious agents such as bacteria, viruses, and parasites [1]. In the early 1950s, Ravich et al. first proposed the infection hypothesis for prostate cancer (PCa) and the potential of prophylaxis [2]. Since then, multiple studies have investigated the association between sexually transmitted infection (STI) and incidence of PCa. A meta-analysis of 6022 cases of prostate cancer and 7320 controls from 29 case–control studies demonstrated a significant association of PCa risk with infection history of any STI-related agent, including *Neisseria gonorrhoeae* and human papillomavirus (HPV) [3]. The evidence from previous studies is limited because most of the previous studies utilized serology-based case–control designs (sero-epidemiology) and the results were inconsistent across the studies. The sensitivity of serological assays is generally low, and seroconversion may take a long time or may not occur in the case of every infectious agent [4]. Moreover, seropositivity to targeted infectious agents does not necessarily reflect the local infection in the prostate tissue of those agents.

Carcinogenesis is associated with multiple factors including age, race, diet, heredity, environment, and inflammation [5]. Over the decades, there has been an interest in understanding the vital role of inflammation in the initiation and progression of PCa. A prospective study linked with two large PCa prevention trial (PCPT and SELECT) cohorts demonstrated that benign tissue inflammation in the baseline biopsy cores was positively associated with a future diagnosis of PCa [6]. Proliferative inflammatory atrophy (PIA) is one of the putative precancerous lesions in which inflammation stress can drive prostate carcinogenesis via the generation of reactive oxygen species, epigenetic alterations, and subsequent mutagenesis [7]. Frequent exposure of the prostate to numerous microorganisms through the urethra may contribute to prostatic infection, formation of an inflammatory microenvironment, and chronic inflammation. Two recent studies have investigated the microbiome of the prostate microenvironment using 16s rRNA gene amplification followed by massive sequencing to identify the specific microbiota or bacteria associated with prostate carcinogenesis [8,9]. However, the evidence from these two studies is limited because they lack a detailed evaluation of intraprostatic inflammation, detection of non-bacterial microorganisms such as mycoplasmas and virus, and sufficient number of samples.

In this study, we examined prostatectomy specimens obtained by radical prostatectomy and corresponding needle biopsy specimens from indolent and aggressive PCa. We screened the clinical samples against a panel of STI-related organisms and pathologic severity of prostatic inflammation to seek the potential association of prostatic infection, inflammatory environment, and prostate carcinogenesis.

## 2. Methods

### 2.1. Patient Selection and Data Collection

**Figure 1:**
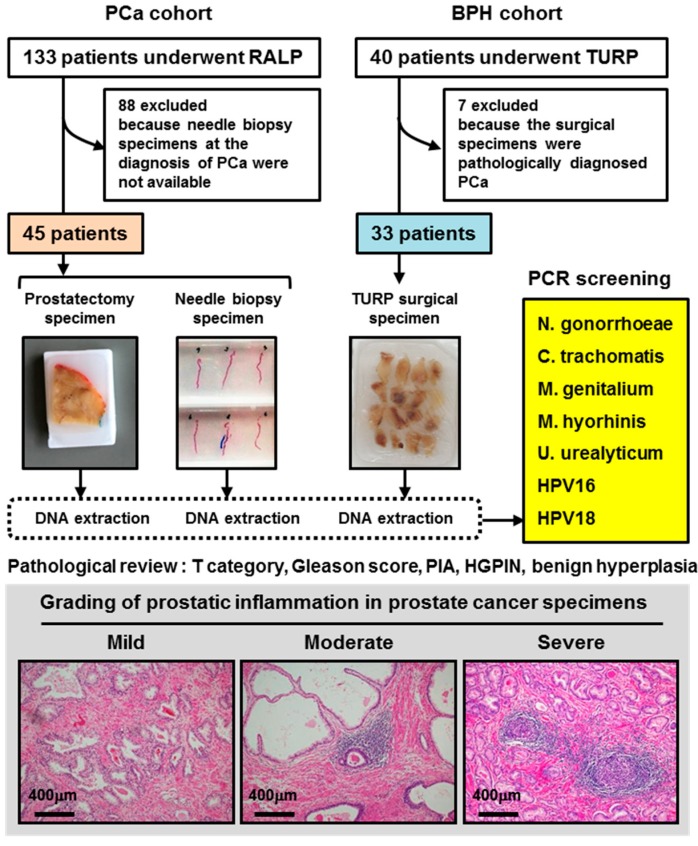
Flow chart of the study design. Intraprostatic inflammation was graded based on the typical inflammatory cell density into three categories: mild, moderate, and severe [11,12]. Abbreviations: PCa: prostate cancer, RALP: robot-assisted laparoscopic radical prostatectomy, BPH: benign prostate hyperplasia, TURP: transurethral resection of the prostate, PCR: polymerase chain reaction, HPV: human papillomavirus, PIA: proliferative inflammatory atrophy, and HGPIN: high-grade prostatic intraepithelial neoplasia.

All subjects gave their informed consent for inclusion before they participated in the study. The study was conducted in accordance with the Declaration of Helsinki, and the protocol was approved by the Ethics Committee of the Nara Medical University (Project identification codes: 1368 and 1966). A total of 133 patients with PCa underwent robot-assisted laparoscopic radical prostatectomy (RALP), and a total of 40 patients with benign prostatic hyperplasia underwent transurethral resection of the prostate (TURP) at the same institute between January 2016 and June 2018. According to the study eligibility, 45 (34%) and 33 (83%) patients were enrolled, respectively (Figure 1). Of the 45 eligible patients undergoing RALP, conventional transrectal needle biopsy was performed in 34 (76%) and transperineal template-guided saturation biopsy was performed in 11 (24%) [10]. An automated urine flow cytometer, Sysmex UF-1000i (Sysmex Medical Electronics Co., Kobe, Japan), was used to detect white blood cells in preoperative urine. The preoperative urinary function was determined by the International Prognostic Scoring System (IPSS) questionnaire.

### 2.2. Pathological Review of Prostate Cancer Specimens

All hematoxylin and eosin-stained (H&E) specimens obtained through surgery or needle biopsy were reviewed by two experienced uropathologists (T.F. and N.T.) for the T category (2010, the 7th edition American Joint Committee on Cancer TNM Staging system), Gleason score (2005, International Society of Urological Pathology classification), presence of PIA, presence of high-grade prostatic intraepithelial neoplasia (HGPIN), and benign hyperplasia (glandular, stromal, or mixed hyperplasia). Prostatic inflammation was graded based on typical inflammatory cell density as previously described [11,12]: mild = individual inflammatory cells, most of which were separated by distinct intervening spaces (<100 cells/mm^2^); moderate = confluent sheets of inflammatory cells with no tissue destruction or lymphoid nodule/follicle formation (100–500 cells/mm^2^); and severe = confluent sheets of inflammatory cells with tissue destruction or nodule/follicle formation (>500 cells/mm^2^). Chronic inflammation predominantly involved lymphocytes, plasma cells, and macrophages. The grading of inflammation was carried out separately for peri-tumoral and non-tumoral areas. When multiple tumoral foci were present, the representative tumoral area was selected according to the highest Gleason score.

### 2.3. DNA Isolation from Prostate Specimens

Total genomic DNA was purified from the formalin-fixed paraffin-embedded specimens using NucleoSpin^®^ DNA FFPE XS (Macherey-Nagel, Düren, Germany) following the manufacturer’s instructions. Briefly, up to seven 10 µm-thick sections of 250 mm^2^ total area (<15 mg paraffin) were subjected to dissolution in paraffin, followed by homogenization and enzymatic digestion with proteinase K. The lysate was further purified using a silica–membrane column to obtain high-quality DNA for polymerase chain reaction (PCR). The concentration and quality of DNA were assessed by spectrophotometry (Bio Spec-nano, Shimadzu, Kyoto, Japan) at 260 and 280 nm. Adequacy of the DNA was confirmed by amplification of a 60 bp region of human beta-actin gene. The isolated DNA was stored at −80 °C prior to PCR.

### 2.4. PCR Screening of Infectious Agents

Primers were synthesized by GeneDesign, Inc. (Osaka, Japan) and screened for *N. gonorrhoeae*, *Chlamydia trachomatis*, *Mycoplasma genitalium* (Mg) [13,14], *Mycoplasma hyorhinis* (Mh) [14], *Ureaplasma urealyticum* [13], HPV16 [15], and HPV18 [15], with minor modifications (Table 1). PCR was performed using a DNA Engine Tetrad 2 thermal cycler (Bio-Rad, Hercules, CA) in a 20 μL reaction mixture, containing max 100 ng of DNA and 200 nmol/L of each primer, along with EmeraldAmp PCR Master Mix (Takara Bio Inc., Shiga, Japan). The amplification steps comprised initial denaturation at 94 °C for 3 min, followed by 40 cycles of denaturation at 95 °C for 30 s, annealing for 1 min, and elongation at 72 °C for 1 min. *Neisseria gonorrhoeae* DNA (Cat# MBC075; Vircell, Granada, Spain), *C. trachomatis* DNA (Cat# MBC012; Vircell), DNA of Ca Ski cells (positive for HPV16 ), DNA of HeLa cells (positive for HPV18), and DNA of Mg/Mh (both maintained in the Hawaii Cancer Center) were used as positive controls for each PCR amplification. The amplified PCR products were electrophoresed on 1% or 3% agarose gel with 100 bp or 50 bp ladder markers, respectively.

**Figure 2:**
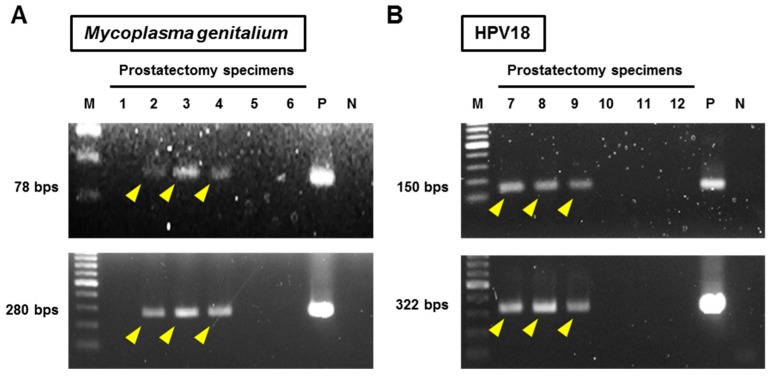
Representative images of PCR for detection of *Mycoplasma genitalium* and human papillomavirus-18. Patient IDs (1–12) are indicated at the top. (**A**) DNA templates were amplified using two primers (78 bp-amplicon and 280 bp-amplicon) targeting *Mycoplasma genitalium*, and the PCR products were electrophoresed on agarose gel with ladder markers. Both the primers were defined as positive for Mg (yellow arrowheads). Patients 2–4 were determined positive for Mg. (**B**) Nested PCRs were performed for HPV detection using MY09/MY11 as the outer and GP5+/GP6+ as the inner primers (upper panel). Type-specific PCR to detect HPV18 was performed (lower panel). Both nested PCR and type-specific PCR were defined as positive for HPVs. Patients 7–9 were determined as positive for HPV18. Abbreviations: M: ladder marker, P: positive control, N: negative control, and HPV: human papillomavirus.

Specifically, two different primer pairs (short amplicon and long amplicon) were used for detection of Mg to increase the specificity. Both the primers were defined as positive for Mg (Figure 2A). Nested PCRs were performed for HPV detection using MY09/MY11 as outer and GP5+/GP6+ as inner primers. One microliter of the MY09/MY11 PCR amplicon was used as the template for the nested PCR amplification with GP5+/GP6+ (25 cycles of denaturation, annealing, and elongation). Subsequently, type-specific PCR to detect the most common high-risk HPV subtypes, HPV16 and HPV18, was performed. Both nested PCR and type-specific PCR were defined as positive for HPV16 and HPV18 (Figure 2B). 

### 2.5. Statistical Analysis

The values are expressed as mean ± standard deviation (SD) and median with range. The values were compared using chi-square test or Mann-Whitney U test. PRISM software version 7.00 (GraphPad Software, Inc., San Diego, CA, USA) was used for statistical analysis and plotting of graphs. *P* values < 0.05 were considered statistically significant.

## 3. Results

### 3.1. Infection Status of Prostate Surgical Specimens

**Figure 3:**
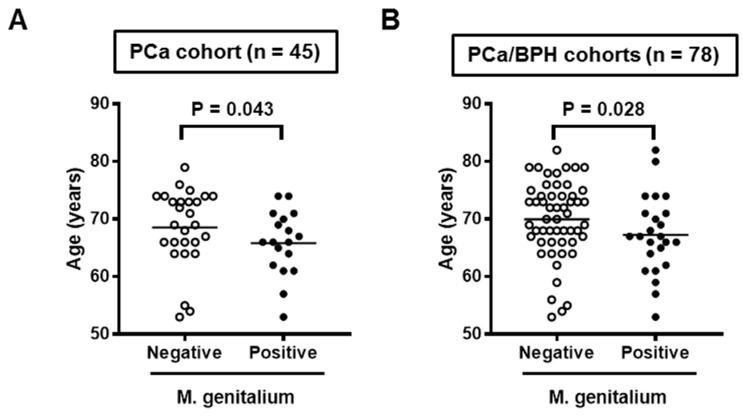
Association between age and *Mycoplasma genitalium* infection status in surgical specimens. Data for patient age at surgery for the *Mycoplasma genitalium*-positive group and negative group are shown as scatterplots in the analysis of the PCa cohort (**A**) and the PCa/BPH cohort (**B**). Mann–Whitney U-test was used to compare the two groups.

A total of 78 surgical specimens, including 45 of RALP and 33 of TURP, were screened for infection by seven STI-related organisms (Table 2). *N. gonorrhoeae*, *C. trachomatis*, *U. urealyticum*, and Mh were not detected in any of the prostate specimens screened in this study. The positive rate of Mg was significantly different between the PCa cohort (18/45, 40%) and the benign prostate hyperplasia (BPH) cohort (6/33, 18%). HPV18 DNA was detected in five (11%) of PCa cohort and two (6%) of BPH cohort, while only one patient of the PCa cohort was positive for HPV16. Out of six PCa patients positive for HPV16/18, four (67%) were positive for Mg, indicating a high rate of mixed infection. With regard to age, the PCa cohort was significantly younger than the BPH cohort (67.5 vs. 71.4 years, *P* = 0.01). Positive status of Mg in surgical specimens was associated with a lower age in the analysis of the PCa cohort (N = 45, Figure 3A) and PCa/BPH cohorts (N = 78, Figure 3B).

### 3.2. Mg Infection, Patient Background, and Prostate Inflammation in the PCa Cohort

Clinicopathological variables of the 45 patients with PCa are listed and the statistical comparison by Mg infection status is shown in Table 3. There was no difference in the count of white blood cells in preoperative urine, preoperative total IPSS score, hypertension, diabetes mellitus, initial prostate-specific antigen (PSA) level, and Gleason score between the Mg-positive and Mg-negative patients, whereas the rate of extensive disease (pT2c–3b) was higher in Mg-positive patients than in Mg-negative patients (77% vs. 44%, *P* = 0.027). No significant correlation was observed between the Mg infection status and grade of intraprostatic inflammation. With regard to precancerous pathologic findings, only 1 and 2 out of 45 patients with PCa presented PIA and HGPIN, respectively. The statistical power for evaluating the clinical impact of precancerous pathologic findings with Mg infection was insufficient because of the extremely small sample size.

### 3.3. Detection Feasibility of Mg and HPVs from Prostate Needle Biopsy Specimens

**Figure 4:**
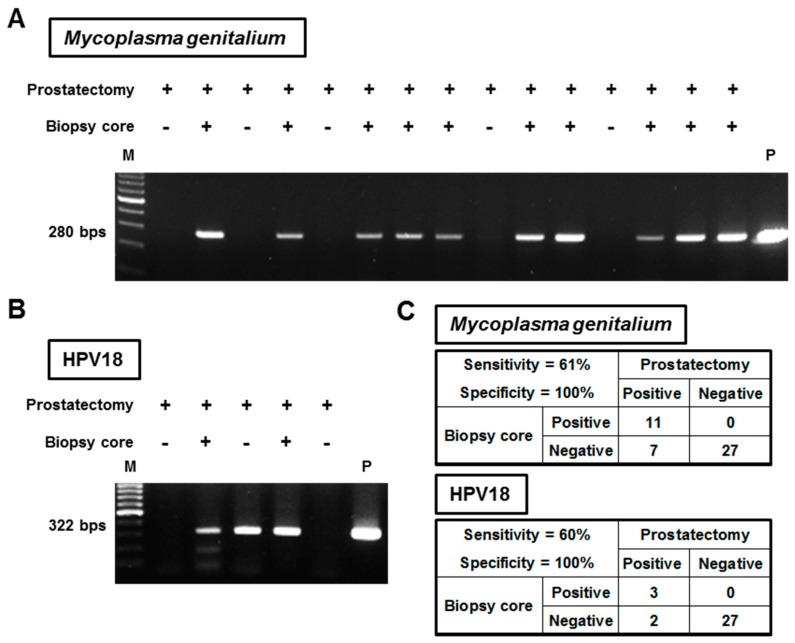
Detection feasibility of *Mycoplasma genitalium* and HPV18 from prostate needle biopsy specimens. (**A**) DNA isolated from prostate needle biopsy specimens was amplified using *Mycoplasma genitalium*-targeting primers, and the PCR products were electrophoresed on agarose gel with ladder markers. All 15 patients shown in this representative image tested positive for *Mycoplasma genitalium* in the prostatectomy specimens. (**B**) Type-specific PCR was performed to detect HPV18 in the needle biopsy specimens. All five patients shown in this representative image tested positive for HPV18 in the prostatectomy specimens. Abbreviations: M: ladder marker, P: positive control, and HPV: human papillomavirus. (**C**) The diagnostic accuracy of needle biopsy specimens for detecting *Mycoplasma genitalium* and HPV18 are tabulated separately.

To examine the detection feasibility of infectious agents from prostate needle biopsy cores, a total of 45 biopsy specimens were screened for infection of Mg, HPV16, and HPV18. *N. gonorrhoeae*, *C. trachomatis*, *U. urealyticum*, and Mh were excluded from this analysis because all prostatectomy specimens were negative for all of these infectious agents. Figure 4A,B shows representative images of screening for Mg and HPV18, respectively. Figure 4C summarizes the detection accuracy of biopsy specimens, showing 61% of sensitivity (11 of 18) for Mg and 60% (3 of 5) for HPV18, and 100% of specificity for both agents. In the analysis of one HPV16-positive patient, HPV16 DNA was not detected from the biopsy specimen.

## 4. Discussion

In the present study, we investigated the prevalence of Mg and HPV infection in the PCa and BPH specimens. The rate of Mg infection in the PCa cohort (40%) was higher than that in the BPH cohort (18%). The additional analysis with age indicated that Mg prostate infection was likely associated with younger patients (Figure 3). Clinically, Mg is one of the most important sexually transmitted pathogens, which can cause several human inflammatory diseases such as urethritis and chronic prostatitis in men, and cervicitis, endometritis, salpingitis, tubal factor infertility, and pelvic inflammatory disease in women [16]. The higher rate of Mg infection in the younger population may be explained by higher sexual activity. Interestingly, four out of six PCa patients with HPV16/18 infection in the prostate were also positive for Mg (Table 2). Mixed infection of HPV and urogenital mycoplasmas has been reported in the cervical smears of women diagnosed with abnormal cervical cytology of various grades [17]. The risk of HPV infection was two-fold higher in patients infected with any of the four analyzed species (Mg, *M. hominis*, *Ureaplasma urealyticum*, and *Ureaplasma parvum*) than in the absence of infection. 

Many studies have investigated the association between carcinogenesis and infection of mycoplasma species such as Mg, Mh, *M. penetrans*, *M. hominis*, and *M. salivarium* [14,18,19,20]. These mycoplasmas are the most detected species in patients with malignancies. However, compared with other microorganisms, the number of studies aimed towards the detection of Mg in cancer patients and understanding the biological role of Mg in cancer progression are still few. Namiki et al. demonstrated persistent Mg infection (more than four months) acquired oncogenic transformation in benign prostatic cells, confirmed by elevated migration/invasion capability, anchorage-independent growth, and tumorigenicity in immunodeficient mice [14]. These changes were associated with an increase in karyotypic entropy, evident by the accumulation of chromosomal aberrations and polysomy. This finding supports the evidence that Mg is able to induce tumorigenesis not only indirectly through a local chronic progressive inflammatory response, but also directly through bacterial protein products such as p37 that exert oncogenic effects [21,22]. Another study by Feng et al. indicated that Mg could inhibit cell apoptosis in a murine myeloid cell line [23]. According to our clinical data (Table 3), the rate of extensive disease (pT2c–3b) was higher in Mg-positive patients (77%) than in Mg-negative patients (44%). Mg infection in a cancer microenvironment could increase the proliferation and anti-apoptotic ability of PCa cells. Subjects in this study include only operable PCa patients. Future studies should focus on the association between Mg infection and advanced metastatic PCa (stage D1 or D2). Another important question for future research is whether Mg infection status affects the clinical outcomes such as postoperative recurrence rate and sensitivity to radiotherapy and/or androgen deprivation therapy.

Chronic prostatic inflammation is frequently observed during and serves as a putative risk factor for PCa initiation and progression. Prostatic infection by potentially pathogenic microorganisms may create an inflammatory microenvironment. We examined the association between Mg infection status and pathologic severity of prostatic inflammation, and observed no significant correlation (Table 3). A recent study addressing the microbiome of the PCa microenvironment reported *Propionibacterium* spp. as the most abundant genera in the prostate microenvironment and *Staphylococcus* spp. as a more represented genera in the tumor/peri-tumoral area. However, they did not find a significant correlation of the microbial burden with the grade of intraprostatic inflammation or inflammatory cell density. As chronic prostatic inflammation is a multifactorial event, it may be difficult to identify one or two microorganisms responsible for the inflammation. There is substantial evidence for the possible association between PCa risk and gene polymorphism involved in the local host response to infectious agents. Polymorphisms in RNASEL [24], COX-2 [25], and TLR4 [26] were reported to be functionally linked with inflammation and immunity, likely suggesting an increased PCa risk.

Yu et al. analyzed the microbiota in urine, expressed prostatic secretion (EPS), and seminal fluid samples from patients with PCa or BPH [27]. The authors successfully detected the presence of diverse bacteria from all three materials and found a significant difference in the microbial population between PCa and BPH. The greatest significance of this study was perhaps that urogenital microbiota could be tested non-invasively from biological fluids. However, when testing for prostate-specific microbiota, possible contaminations by urothelium and glans could be a problem. Prostate needle biopsy core could be free from those contaminations, enabling a less invasive determination of prostate-specific microbiota. We examined the detection feasibility of infectious agents from prostate needle biopsy cores. The detection sensitivity of biopsy specimens was 61% for Mg and 60% for HPV18. There may be some possible clinical applications of testing Mg or HPVs, as described below. For example, a patient receiving prostate needle biopsy has Mg-positive cores without a pathological detection of PCa. Given that the positivity of Mg infection is associated with the risk of PCa, the risk of future diagnosis of PCa would be high. Thus, early re-biopsy or close follow-up should be recommended for such patients. Another possible intervention would be the targeting of infectious agents by antibiotics, similar to the treatment of *Helicobacter pylori* [28].

The present study has several limitations. First, there may be a selection bias, for example, some patients were excluded due to the unavailability of biopsy materials. Second, we analyzed only two high-risk HPVs (16 and 18). There are 11 types of high-risk HPVs (HPV16, 18, 31, 33, 35, 39, 45, 52, 56, 58, and 68) reported to be associated with anogenital lesions [29]. Moreover, our result showed only six patients with HPV16/18 infection in prostate cancer tissues, which could not interpret anything. Third, we utilized formalin-fixed paraffin-embedded specimens and a qualitative approach targeted towards single microorganisms, which was limited by both sensitivity and specificity. Finally, the analysis included only a small number of patients and did not include the assessment of outcomes due to the short follow-up duration after RALP.

In conclusion, a comprehensive understanding of the correlation between the urogenital microbiome and chronic prostatic inflammation could facilitate the development of novel strategies for PCa prevention.

## Figures and Tables

**Table 1:** Primers used in this study for detection of infection agents in prostate specimens.

Target Organism	Primer Sequence (5’ to 3’)		Annealing T (°C)	Product Size (bps)
***Neisseria gonorrhoeae***	Forward	GCGACGTCATCGGTAAATACC	60	68
	Reverse	CGCCATACGGACGATGGT		
***Chlamydia trachomatis***	Forward	TGATGTATCCAGCCCAAATGC	50	81
	Reverse	AATCCAGTTCTTCTCTGCCTCTCTAC		
***Mycoplasma genitalium* (short amplicon)**	Forward	GAGAAATACCTTGATGGTCAGCAA	60	78
	Reverse	GTTAATATCATATAAAGCTCTACCGTTGTTATC		
***Mycoplasma genitalium* (long amplicon)**	Forward	AGTTGATGAAACCTTAACCCCTTGG	60	280
	Reverse	CCGTTGAGGGGTTTTCCATTTTTGC		
***Mycoplasma hyorhinis***	Forward	GATCACATTTCCACTTATTTGAAACA	60	460
	Reverse	AAACGACGTCCATAAGCAACTTTA		
***Ureaplasma urealyticum***	Forward	ACACCATGGGAGCTGGTAAT	60	100
	Reverse	CTTCTCGACTTTCAGA		
**HPV MY09/MY11**	Forward	CGTCCMARRGGAWACTGATC	60	450
	Reverse	GCMCAGGGWCATAAYAATGG		
**HPV GP5+/GP6+**	Forward	TTTGTTACTGTGGTAGATACTAC	50	150
	Reverse	GAAAAATAAACTGTAAATCATAT		
**HPV16**	Forward	CACAGTTATGCACAGAGCTGC	60	457
	Reverse	CATATATTCATGCAATGTAGGTGTA		
**HPV18**	Forward	CACTTCACTGCAAGACATAGA	60	322
	Reverse	GTTGTGAAATCGTCGTTTTTCA		
**human b-actin**	Forward	TGAGCGCGGCTACAGCTT	60	60
	Reverse	TCCTTAATGTCACGCACGATTT		

HPV, human papillomavirus.

**Table 2:** Infectious organisms in prostate surgical specimens.

Target Organism	PCa (RALP) n = 45	BPH (TURP) n = 33	*P* Value
**Age (years old) at Surgery**	67.5 ± 6.3	71.4 ± 6.9	0.01
***Neisseria gonorrhoeae***	0	0	NA
***Chlamydia trachomatis***	0	0	NA
***Mycoplasma genitalium***	18 (40%)	6 (18%)	0.03
***Mycoplasma hyorhinis***	0	0	NA
***Ureaplasma urealyticum***	0	0	NA
**HPV16**	1 (2%) ^†^	0	0.39
**HPV18**	5 (11%) ^†^	2 (6%)	0.44

NA, not available; †, Out of six patients with HPVs, four were positive for *Mycoplasma genitalium*.

**Table 3:** Clinicopathological variables of the 45 patients with PCa and the statistical comparison by Mycoplasma genitalium infection status in prostate surgical specimens.

Variables		Total (n = 45)	*Mycoplasma genitalium*		*P* Value
			Negative (n = 27)	Positive (n = 18)	
**Age (years) at RALP**	mean ± SD	67.5 ± 6.3	68.6 ± 6.7	65.8 ± 5.6	0.043
	median (range)	68 (53–79)	68 (53–79)	66 (53–74)	
**initial PSA (ng/mL)**	mean ± SD	13.3 ± 13.1	13.2 ± 12.1	13.5 ± 14.8	0.85
	median (range)	7.6 (3.8–61.3)	7.6 (3.8–48.8)	6.9 (4.2–61.3)	
**Preoperative urine WBC (/mL)**	mean ± SD	11.9 ± 34.2	13.2 ± 39.6	9.6 ± 22.4	0.93
	median (range)	1.9 (0.3–199)	1.9 (1.3–199)	1.9 (0.3–88.6)	
**Preoperative IPSS**	mean ± SD	11.0 ± 7.6	10.5 ± 6.7	11.7 ± 8.7	0.93
	median (range)	10 (1–31)	9 (1–26)	11 (2–31)	
**Hypertention**	No	24 (53%)	17 (63%)	7 (39%)	0.11
	Yes	21 (47%)	10 (37%)	11 (61%)	
**Diabetes mellitus**	No	39 (87%)	24 (89%)	15 (83%)	0.59
	Yes	6 (13%)	3 (11%)	3 (17%)	
**Prostatectomy Gleason score**	3 + 4	19 (42%)	10 (37%)	9 (50%)	0.68
	4 + 3	18 (40%)	12 (44%)	6 (33%)	
	4 + 4/4 + 5	8 (18%)	5 (19%)	3 (17%)	
**Prostatectomy T category**	pT2a	15 (33%)	12 (44%)	3 (17%)	0.027 ^†^
	pT2b	4 (9%)	3 (11%)	1 (6%)	
	pT2c	12 (27%)	4 (15%)	8 (44%)	
	pT3a	10 (22%)	5 (19%)	5 (28%)	
	pT3b	4 (9%)	3 (11%)	1 (6%)	
**Inflammation of peri-tumoral area**	None	17 (38%)	10 (37%)	7 (39%)	0.58
	Mild	23 (51%)	15 (56%)	8 (44%)	
	Moderate	2 (4%)	2 (7%)	2 (11%)	
	Severe	1 (2%)	0 (0%)	1 (6%)	
**Inflammation of non-tumoral area**	None	14 (31%)	11 (41%)	3 (17%)	0.21
	Mild	23 (51%)	13 (48%)	10 (56%)	
	Moderate	7 (16%)	3 (11%)	4 (22%)	
	Severe	1 (2%)	0 (0%)	1 (6%)	
**PIA**	No	44 (98%)	26 (96%)	18 (100%)	0.82
	Present	1 (2%)	1 (4%)	0 (0%)	
**HGPIN**	No	43 (96%)	25 (93%)	18 (100%)	0.23
	Present	2 (4%)	2 (7%)	0 (0%)	

SD, standard deviation; PSA, prostate-specific antigen; WBC, white blood cell; IPSS, International Prognostic Scoring System; ^†^, comparison between pT2a-b vs pT2c-3b.
